# Food environment and fruit and vegetable intake in a urban population: A multilevel analysis

**DOI:** 10.1186/s12889-015-2277-1

**Published:** 2015-10-05

**Authors:** Milene Cristine Pessoa, Larissa Loures Mendes, Crizian Saar Gomes, Paula Andréa Martins, Gustavo Velasquez-Melendez

**Affiliations:** Departamento de Nutrição e Saúde, Universidade Federal de Viçosa, Viçosa, Minas Gerais Brazil; Departamento de Nutrição, Universidade Federal de Juiz de Fora, Juiz de Fora, Minas Gerais Brazil; Departamento de Enfermagem Materno-Infantil e Saúde Pública, Escola de Enfermagem, Universidade Federal de Minas Gerais, Av. Alfredo Balena, 190, Belo Horizonte, Minas Gerais Brasil 30130-100; Departamento de Ciências do Movimento Humano, Universidade Federal de São Paulo, Santos, São Paulo Brazil

**Keywords:** Food environment, Fruit and vegetable consumption, Surveillance system, Multilevel analysis

## Abstract

**Background:**

Environmental, social and individual factors influence eating patterns, which in turn affect the risk of many chronic diseases. This study aimed to estimate associations between environmental factors and the consumption of fruit and vegetables among adults in a Brazilian urban context.

**Methods:**

Data from the surveillance system for risk factors for chronic diseases (VIGITEL) of Brazilian Ministry of Health were used. A cross-sectional telephone survey (VIGITEL – 2008–2010) was carried out with 5826 adults in the urban area of Belo Horizonte. Individual variables were collected. The frequency of fruit and vegetables consumption was assessed from number of servings, weekly frequency and an intake score was calculated. Georeferenced variables were used to characterize the food environment. The density of healthy food outlets (stores specialized in selling fruit and vegetables), unhealthy food outlets (bars, snack bars and food trucks/trailers) and the neighborhood family income were investigated and associated with fruit and vegetables intake score. Weighted multilevel linear regression was used to evaluate the associations between the environment variables and the fruit and vegetables intake score.

**Results:**

Higher fruit and vegetables intake scores were observed in neighborhoods with higher density of healthy food outlets and higher income. Lower scores were observed in neighborhood with higher density of unhealthy food outlets. These associations were adjusted by individual variables such as gender, age, physical activity, sugar sweetened beverages consumption, education level and smoking.

**Discussion:**

The food environment might explain some of the socioeconomic disparities with respect to healthy food intake and health outcomes. Healthy food stores are less common in socially disadvantaged neighborhoods, and therefore, healthy foods such as fruits and vegetables are less available or are of a lower quality in lower income areas.

**Conclusion:**

Food environment characteristics and neighborhood socioeconomic level had significant associations with fruit and vegetable intake score. These are initial findings that require further investigation within the middle income world populations and the role of the environment with respect to both healthy and unhealthy food acquisition and intake.

## Background

Fruit and vegetable intake has been associated with a reduced risk of mortality [1] and occurrence of chronic diseases, such as cardiovascular disease [2], stroke [3], and some types of cancer [4]. A World Health Organization (WHO) publication stated convincing evidence that fruit and vegetable intake also reduces the risk of diabetes and obesity [5]. Fruit and vegetable intake is characteristic of healthy eating patterns, which is related to lower risk of non-communicable chronic disease (NCCD) prevention. Despite the human health-related benefits of fruit and vegetable intake, a low prevalence of intake has been observed worldwide [6]. In Brazil, this was shown in the most recent national survey, an insufficient intake of fruit and vegetable in the diets among all socioeconomic levels [7].

Although the increased intake of fruits and vegetables is a priority action on the agenda of the National Health Promotion Policy in Brazil [8], the implementation has many difficulties that are most likely due low knowledge regarding the factors associated to health dietary practices [9].

Previous studies have shown that important socioeconomic factors, such as a low family income and educational level, are related to low fruit and vegetable intake [7, 10–12]. However, the determinants of dietary intake do not include only individual characteristics.

In the late 1990s, Swinburn, Egger and Raza [13] proposed a causal ecological model of obesity that could be evaluated according to the built environment, which included elements of urban design, land use, public transportation, physical activity options and healthy food availability and access, all of which promote healthy or unhealthy behaviors [13].

Regarding obesity, researchers also have used other conceptual models of food intake patterns and determinants [12, 14, 15]. One of the theoretical model for food intake patterns developed by Glanz et al. [15] identified 4 categories of variables: those related to political issues, the environment, the individual and behaviors [15].

Story et al. [12] proposed another ecological model which dietary behaviors are highly complex [12] because they result from the interaction of multiple influences in different contexts [13, 16]. This model considered the individual level, the social environment, the built environment and the macroenvironment [12].

In general, studies show that availability and access to food stores and food prices are environmental factors that influence food consumption of FV. In addition to the food environment, economic conditions of the neighborhoods are also associated with FV consumption [12, 15]. It is observed that most economically favored neighborhoods present greater availability of healthy food stores and the foods have superior quality that in worst conditions neighborhoods [17, 18].

In this context, in recent years, several studies have hypothesized roles of the environment on health outcomes carried out using an ecological approach which could better explain population's health behaviors [19, 20]. Although food environment have been extensively studied in developed countries [21–24] the theme needs to be explored in greater depth in Brazilian studies with the use of different methodologies, populations (children, adolescents, adults, and the elderly), and contexts (households, schools, and the workplace). Historically Brazilian cities are characterized by process of socio spatial segregation produced by social inequities that affect the offer of public services in the poor neighborhoods.

This study aimed to explore the associations between environmental variables and fruit and vegetables intake scores in adult urban population of a state Brazilian capital, during the years of 2008 to 2010.

## Methods

### Study sampling and population

This study was conducted using data collected from individuals living in the city of Belo Horizonte, the state capital of Minas Gerais, Brazil. This city is located in southeastern Brazil and covers a total area of 331 km^2^; the city has a population of 2,365,151 inhabitants and a population density of 7,177 inhabitants/km^2^ [25].

Data were collected using a standardized questionnaire during telephone interviews with individuals living in the city of Belo Horizonte, aged 18 years or older, conducted by the Telephone Surveillance System for Risks and Protective Factors for Chronic Non-communicable Diseases (VIGITEL). The questionnaire requested self-reported information regarding sociodemographic characteristics, dietary patterns, weight, height, physical activity and health self-perception variables. The sampling procedures used by the VIGITEL system aimed to establish annual probabilistic samples of the adult population in each Brazilian capital living in households with at least 1 fixed telephone line [26].

The VIGITEL system did not employ a direct method to compensate for the fraction of households not served by telephone in each city or in each population stratum. However, post-stratification weights were assigned to the individuals interviewed by the system to at least partially correct for the biases created by the lack of universal telephone network coverage [26].

A total of 6,034 interviews, realized in 2008, 2009 and 2010, were considered eligible for this study; of these interviews, 5,826 (96.55 %) were georeferenced, of which 215 lacked information regarding variables related to fruit and vegetable intake. The final sample therefore comprised 5,611 persons with georeferenced information and information regarding fruit and vegetable intake.

### Characterization of environmental/ Contextual variables

The participants’ residences were geocoded using household ZIP codes. Geographic coordinates (latitude and longitude) were obtained using the centroid of the street corresponding to the ZIP codes.

For this study health administrative areas of the city, called coverage areas (CA), was used as neighborhood unit. Georeferenced information regarding the each CA was obtained from official health sources. Brazilian’s National Health System is organized by CA units, each of them has one Basic Health Unit. The CA are core of health policies and the local government provides basic health care services. Belo Horizonte is divided into 148 CA.

Characteristics of food stores including localization data were obtained according to the National Classification of Economic Activities, which is the instrument for the national standardization of economic activity codes and eligibility criteria used by the various national Tax Administration organs [27]. Data regarding public facilities for municipal food safety and nutrition policy were also included, such as city-subsidized open-air markets specialized in selling fruits and vegetables and restaurants with popular prices that focused on offering nutritionally balanced prepared meals. The geocoding process was based on the addresses and postal codes of all of these locations.

Data such as the sum of the nominal monthly income of persons aged 10 years or older in the CA, termed the "overall income of the CA," and the total, male and female populations were obtained from the Brazilian Institute of Geography and Statistics databases [25].

The mean CA size was 2.55 km^2^; the smallest and largest CA were 0.31 km^2^ and 14.69 km^2^, respectively. An average of 7000 households and 21,000 inhabitants were observed in each CA. The mean number of study participants per CA was 37.9, with a range of 2–165 participants per area.

### Dependent variable

Fruit and vegetable intake was measured by the following questions: "How many days per week do you usually eat at least 1 type of vegetable?", "How many days per week do you usually drink natural fruit juice?" and "How many days per week do you usually eat fruits?" The response options were "1 to 2 days per week," "3 to 4 days per week," "5 to 6 days per week," "every day (including Saturday and Sunday)," "almost never" and "never." Another question evaluated the frequency of fruit intake on an ordinary day, with response options of once, twice or 3 or more times per day. Both of these questions were used to estimate the daily frequency of fruit intake. The vegetable intake frequency on a typical day was evaluated with the following options: at lunch, dinner or at lunch and dinner. The daily vegetable intake frequency was calculated as the sum of the daily frequencies of vegetable, raw salad and cooked vegetable intake. These variables related to food intake in Belo Horizonte, which were investigated by VIGITEL, had been validated in a study by Mendes et al. [28] The fruit and vegetable intake scores were established according to the provided answers. The scores ranged from 0–12, as shown in Table 1, where 0 means that the FV consumption occurred never or almost never and 12 means that the FV consumption occurred every day, at least 2 times a day.

**Table 1:** Calculation of score of fruits and vegetables intake

Score	0	1	2	3	4
Fruits	Never/Almost never	1-2 times/week	3-4 times/week	Every day or 5–6 times/week and 1–2 times/day	Every day or 5-6x times/week and ≥3 times/day
Raw salad	Never/Almost never	1-2 times/week	3-4 times/week	Every day or 5–6 times/week and 1 time/day	Every day or 5–6 times/week and 2 times/day
Cooked Vegetables	Never/Almost never	1-2 times/week	3-4 times/week	Every day or 5–6 times/week and 1 time/day	Every day or 5–6 times/week and 2 times/day

Note: Adapted from Souza *et al.* (2011) [42]

### Independent variables

The independent variables in this study were based on the literature and selected from the VIGITEL database and collected georeferenced data. The individual variables were gender, age, leisure time physical activity, smoking, sugar sweetened beverages consumption, education level, skin color, marital status, job, fatty meat consumption and binge drinking.

Census data from 2010 was used to characterize demographic and socio-economic attributes of CA including total monthly income of CA and population density. Additionally, density of supermarkets and hypermarkets, density of mini markets, grocery stores and warehouses, density of healthy food outlets (stores and open-air markets specialized in selling FV), density of restaurants and density of unhealthy food outlets (bars, snack bar and food trucks/trailers) were used. It highlights that unhealthy food outlets sell fast food like hamburgers, hot dogs, pasta, pizza and candies and we did not consider hypermarkets and supermarkets as healthy food outlets because they sell a lot of processed and ultra-processed products.

### Data analysis

The survey module was used for the descriptive analysis of individual and environmental data. This module considered various aspects of the complex sampling design.

To characterize the sample, variable frequencies were calculated according to gender. The means and standard errors of the fruit and vegetable intake scores were also ascertained calculated based on the independent variables. Differences between the categories were evaluated with 95 % confidence intervals. Environmental variables were described using central tendency and dispersion measures.

Multilevel linear regression was used for modeling, considering the hierarchical structures of the data aggregation levels, individual VIGITEL variables and contextual variables for the CA to avoid an underestimation of the standard error values, ascertain the correct confidence intervals and correct the hypothesis testing. A weighted multilevel linear regression analysis (a random intercept model) was performed using the xtmixed function in Stata 12.1 software (StataCorp, College Station, TX, USA). Variables were introduced into the model hierarchically, beginning with the individual variables and followed by the environmental variables. Collinearity was tested before the introduction of the independent variables in the models. A significance level of 0.05 was set for analytical procedures.

### Ethical issues

The VIGITEL project was approved by the National Ethics Committee on Human Research of the Ministry of Health and the Research Ethics Committee of the Federal University of Minas Gerais under protocol no. 25447414.1.0000.5149.

## Results

A total of 5611 individuals were analyzed in this study. The sample comprised 45.2 % men (95 % CI 43.2 – 47.3 %) and 54.8 % women (95 % CI 52.7 – 56.8 %), with a mean age of 39.7 years (95 % CI 39.0 – 40.3 years).

Regarding the response variable of this study (fruit and vegetable intake score), it was observed that in 2008, 2009 and 2010, the mean intake scores were 6.05 (95 % CI 5.85 – 6.24), 6.09 (95 % CI 5.94 – 6.25) and 6.25 (95 % CI 6.11– 6.41), respectively. It was verified that the mean fruit and vegetable intake was higher among women and among more educated people, people aged 65 years or older and among people who were physically active during their leisure time. Lower fruit and vegetable intake scores were observed among people who reported consumption of fatty meat, sugar sweetened beverages intake in 5 or more times a week, among physically inactive people and among those with a poor health self-perception.

Descriptive measures of the environmental variables are presented in Table 2. There was high variability in the mean total income across the CA. High variability was also observed in the mean density of food outlets.

**Table 2:** Description of the environmental variables according to the coverage areas in Belo Horizonte, MG

Variables	Mean	Standard Error	Median	Minimum	Maximum
Total monthly income of CA (in Reals)	34.2 million	42.3 million	15.7 million	1.4238 million	204 million
Population density (inhabitants/km^2^)	9706.68	3719.58	9530.25	519.44	26165.25
Density* of supermarkets and hypermarkets	1.38	0.02	1.25	0	7.68
Density* of mini markets and grocery stores and warehouses	7.47	0.06	6.78	0	22.72
Density* of healthy food outlets	3.04	0.03	2.76	0	19.64
Density* of restaurants	16.75	0.33	8.08	0	117.12
Density* of unhealthy food outlets	36.06	0.07	25.94	0	185.70

Healthy food outlets = stores and open-air markets specialized in selling fruits and vegetables

Unhealthy food outlets = bars, snack bar and food trucks/trailers

*Density = number of food stores/ area of CA in Km^2^

Table 3 shows the multilevel linear regression model without explanatory variables. The variance of the intercept (0.48) demonstrated that this mean differed across the CA, whereas the Wald test showed that this variance was nonzero. The intra-class correlation of 0.09 indicated that 9.0 % of the variability in the individuals' fruit and vegetable intake scores could be attributed to the neighborhood unit. The use of a multilevel approach was justified by this variance value, observed with the intra-class correlation coefficient (ICC) and the p-value of the likelihood-ratio (LR) test. This approach compared the model containing only the fixed effects and a model containing the random effects, the second of which was significant.

**Table 3:** Multilevel linear regression model weighted without explanatory variables - empty model

	Empty model		
Fixed effect	Effect	Standard Error	
Intercept	6.06	0.06	
Random Effect - Level 2 σ^2^_*u0*_			t-ratio*
Variance – intercept	0.48	0.06	8.00
Random Effect - level 1 σ^2^_*e*_			
Variance of *R*_*ij*_	4.68	0.12	39.00
Intra-class correlation coefficient	0.09		

R = residual

LR test p-value <0.001

*Wald test - critical value = 1.96

Table 4 shows the estimated multilevel linear regression model in which explanatory variables were included at the individual and environmental levels. The FV intake scores were higher in women, older people, people who were physically active during leisure time and people with higher education level, but lower in smokers and people who reported consumption of sugar sweetened beverages in 5 or more days a week. Regarding the environment variables, higher scores of FV intake were observed in neighborhood with greater densities of healthy food outlets and higher income. However, lower FV scores were observed in neighborhood with greater density of unhealthy food outlets.

**Table 4:** Final multilevel linear regression model with fruit and vegetable intake score as a response variable

Variables				
Fixed effect	Effect	SE	CI 95 %	*p*- value
Intercept	4.80	1.16	2.51 7.09	<0.001
Male gender (vs. female)	0.59	0.10	0.39 – 0.80	<0.001
Age	0.01	0.004	0.002 -0.02	<0.001
Active in LTPA	0.72	0.13	0.47 – 0.97	<0.001
Smoking (yes vs. no)	−0.29	0.12	−0.52 - -0.05	0.018
SSB intake (5 or more days per week)	−0.28	0.11	−0.47 - -0.09	0.004
Education level (y)				
0-8 (reference)				
9-11	0.68	0.11	0.46 – 0.90	<0.001
12 or more	0.90	0.16	0.57 – 1.21	
Density^1^ of healthy food outlets^3^ in CA^2^	3.93	0.90	2.16 – 5.70	<0.001
Density^1^ of unhealthy food outlets^4^ in CA^2^	−0.88	0.23	−1.32 - -0.43	<0.001
Total income in CA^2^	0.31	0.07	0.17 – 0.46	<0.001
Random effect –level 2 σ^2^_*u0*_				t-ratio*
Variance – Intercept	201.16	165.96	3.47 – 5.78	1.21
Slope variance – gender	1.56	0.24	1.14 – 2.11	6.50
Slope variance – age	0.003	0.001	0.002 – 0.004	3.00
Slope variance – leisure time physical activity	2.34	0.42	1.65 – 3.32	5.57
Slope variance – smoking	2.06	0.29	1.64 – 3.32	7.10
Slope variance – education level (years of study)	0.98	0.16	0.72 – 1.34	6.13
Random effect – level 1 σ^2^_*e*_				
Variances of *R*_*ij*_	3.28	0.12	3.06 – 3.52	27.33

SE – standard error

LR test p-value <0.001

95 % CI = 95 % Confidence Interval

* Wald test - critical value = 1.96

SSB: Sugar sweetened beverages

LTPA: Leisure time physical activity

^1^Density = number of stores/Area in Km^2^

^2^CA: coverage areas by the basic health units

^3^Stores and open-air markets specialized in selling fruit and vegetables

^4^Bars, snack bars and food trucks/trailers

The individual level variables demonstrated a significant random coefficient for level 2 random effect variances, as demonstrated by the Wald test. In other words, the effects of the variables of gender, age, leisure time physical activity, smoking, sugar sweetened beverages consumption and education level were not equal across all CA.

## Discussion

This study revealed that the mean fruit and vegetable intake score of Belo Horizonte population was 6.0, meaning an intake of fruit and vegetables or vegetables 5 or more days a week, with 1–2 servings of fruit and only 1 serving of vegetables daily. This intake can be considered low and has been widely corroborated in national household budget surveys [7].

It was observed high variability in average of total income across the CA, which emphasizes high inequity compared to income in the city. High variability of food outlets density was also showed. Studies conducted in another Brazilian city have shown high income inequities and that food stores tend to aggregate in regions with higher purchasing power. This areas have a higher density of all types of food outlets in addition to better public transport structure and a higher density of parks and public places for the practice of sports [18].

Associations between food and social environment variables and fruit and vegetable intake scores were also estimated in this study. Higher density of healthy food outlets in the neighborhood area was associated to FV intake scores, but higher density of unhealthy food outlets were negatively associated to FV intake scores. Regarding the social environment, higher neighborhood income was correlated with FV intake scores. These associations were significant after adjusted by individual variables such as gender, age, sugar sweetened beverages consumption, education level, physical activity and smoking.

Regarding the individual variables that remained in the multivariate models, fruit and vegetable intake score was higher in women and associated with age and education. Healthy behaviors, evaluated by variables such as leisure time physical activity, were also associated with higher fruit and vegetable intake by individuals. In contrast, unhealthy behaviors, such as smoking and consuming sugar sweetened beverages were associated with a lower fruit and vegetable intake, a finding that have been observed in previous studies [29–32].

Nationally representative studies of Brazilian adult population [33] also showed a positive association between income, education level, age and fruit and vegetable intake. Higher FV intake was observed in people with higher incomes, education levels and ages, a finding that has also been demonstrated in other studies of individuals of both genders [29–32]. Women consumed more fruits and vegetables than men, which has also been demonstrated in international studies [34, 35].

Respecting the environmental variables, the linear multilevel regression model showed a significantly association with the FV intake score in response to the increase of density of stores of fruit and vegetables in the neighborhood. Studies showed the association between increased FV intake and an increased number of stores that sell these types of food, such as supermarkets or stores specializing in the sale of fruit and vegetables [11, 24, 36, 37]. A study conducted in Brazil also found a positive correlation between the neighborhood density of stores specializing in the sale of fruit and vegetables and the intake of such foods [18].

Unhealthy food outlets were negatively associated with FV consumption. The availability of unhealthy food stores in the neighborhood contributes to the obesogenic environment and this is associated with increased consumption of unhealthy products [38]. Studies show that consumption of unhealthy products is related to the low intake of fruits, vegetables and fiber in adults [39, 40].

Other aspects of the factors associated with fruit and vegetable intake, the food environment might explain some of the socioeconomic disparities with respect to healthy food intake and health outcomes. Healthy food stores are less common in socially disadvantaged neighborhoods, and therefore, healthy foods such as fruits and vegetables are less available or are of a lower quality in lower income areas [11].

Findings similar to those obtained in this study were also observed in a study of adult African Americans, Hispanics and non-Hispanic whites living in Detroit, USA. These authors reported that the presence of large neighborhood grocery stores was associated with fruit and vegetable intake, but the distance to the establishment was not associated with intake. Presence of a large grocery store was associated with a increase in average daily fruit and vegetable servings among Latinos compared with African-Americans. Presence of a convenience store was negatively related to fruit and vegetable intake while more stores selling fresh produce was positively related to consumption among Latinos, but not African- Americans [41].

In the present study, total income of the CA area were also found to be an important factor positively associated with fruit and vegetable intake. Material conditions, including the financial situation (e.g., family income), social deprivation, poor working conditions and unfavorable housing and neighborhoods can affect dietary behaviors. An individual’s financial situation can partly determine the access to various products and even to certain food stores, thereby making it difficult to purchase healthy foods [11, 37].

Some limitations should be noted. The first is that despite the use of a causal model; the cross-sectional design did not allow conclusions to be drawn in terms of causality. The second is inherent in the methodological proposal because for practical purposes, in health monitoring systems, the studied dependent variable has a self-reported nature. This problem is minimized, because validation studies conducted with participants of the VIGITEL sample, which demonstrated high correlation rates when obtained measurements were compared with self-reported values and showed good sensitivity and specificity analyses. A third limitation is that the sample was composed of people living in households with fixed telephone lines. However, sample weights were considered during the data analysis to adjust the sample according to the sociodemographic composition of the Brazilian population. It should also be emphasized that an analysis of food prices, the perceived environment and access to stores selling food, rather than just availability—as analyzed in this study—as well as information regarding the entire family or household, would provide more consistent results regarding the association of environmental factors with fruit and vegetable intake. Additionally, more consistent results would be obtained if it were possible to analyze the individual in the context of his/her work and not simply in a residential context, as allowed by data monitoring systems such as VIGITEL.

## Conclusions

This study shows that food and social environment characteristics such as the density of healthy food outlets, neighborhood socioeconomic level and the density of unhealthy food outlets had significant associations, with fruit and vegetable intake scores and that these associations remained after adjusting for individual variables. These are initial findings that require further investigation within the middle income world populations and the role of the environment with respect to both healthy and unhealthy food acquisition and intake.

Monitoring systems such as VIGITEL could be important instruments in this type of research and, with the aid of environmental information, can become important tools for the planning of actions aimed at promoting healthy eating in urban environments.
